# Inactivation of the *htps*A gene affects capsule development and pathogenicity of *Streptococcus suis*

**DOI:** 10.1080/21505594.2020.1792080

**Published:** 2020-08-20

**Authors:** Hua Ni, Min Li, Qiaoqiao Wang, Jing Wang, Xumiao Liu, Feng Zheng, Dan Hu, Xu Yu, Yifang Han, Qi Zhang, Tingting Zhou, Yiwen Wang, Chunhui Wang, Jimin Gao, Zhu-Qing Shao, Xiuzhen Pan

**Affiliations:** a Department of Microbiology, Hua Dong Research Institute for Medicine and Biotechnics, Nanjing, China; b Key Laboratory of Biological Resources and Ecology of Pamirs Plateau in Xinjiang Uygur Autonomous Region, College of Life and Geographic Sciences, Kashi University, Kashi, China; c Clinical Laboratory Department of Changzhi, People’s Hospital, Changzhi, China; d School of Life Sciences, Nanjing Normal University, Nanjing, China; e Department of Laboratory Medicine, The Affiliated Wuxi Maternity and Child Health Care Hospital of Nanjing Medical University, Wuxi, China; f School of Laboratory Medicine and Life Science, Wenzhou Medical University, Wenzhou, China; g State Key Laboratory of Pharmaceutical Biotechnology, School of Life Sciences, Nanjing University, Nanjing, China

**Keywords:** *Streptococcus suis* serotype 2, histidine triad proteins, gene knockout, bacterial virulence, capsule development

## Abstract

Streptococcus suis

serotype 2 (*S. suis* 2) is an important swine pathogen and also an emerging zoonotic agent. HtpsA has been reported as an immunogenic cell surface protein on the bacterium. In the present study, we constructed an isogenic mutant strain of *htps*A, namely Δ*htps*A, to study its role in the development and virulence of *S. suis* 2. Our results showed that the mutant strain lost its typical encapsulated structure with decreased concentrations of sialic acid. Furthermore, the survival rate in whole blood, the anti-phagocytosis by RAW264.7 murine macrophage, and the adherence ability to HEp-2 cells were all significantly affected in the Δ*htps*A. In addition, the deletion of *htps*A sharply attenuated the virulence of *S. suis* 2 in an infection model of mouse. RNA-seq analysis revealed that 126 genes were differentially expressed between the Δ*htps*A and the wild-type strains, including 28 upregulated and 98 downregulated genes. Among the downregulated genes, many were involved in carbohydrate metabolism and synthesis of virulence-associated factors. Taken together, *htps*A was demonstrated to play a role in the morphological development and pathogenesis of the highly virulent *S. suis* 2 05ZYH33 strain.

## Introduction

*Streptococcus suis* (*S. suis*) is an important pathogenic bacterium in swine worldwide and causes a variety of diseases, such as meningitis, endocarditis, septicemia, arthritis, pneumonia, and even acute death [1]. This pathogen can also infect humans via close contact with infected swine or pork-derived products, causing meningitis, endocarditis, septicemia, permanent deafness, and streptococcal toxic shock-like syndrome (STSLS) [2]. Based on the differentiation of capsule antigens, *S. suis* was divided into 35 serotypes, but several recent reports manifested that serotypes 20, 22, 26, 32, 33, and 34 did not belong to the *S. suis* taxon [3–5]. Among all serotypes, *S. suis* 2 is the most virulent and frequently isolated serotype from clinically diseased piglets [6,7]. During the last 20 years, sporadic or large outbreaks of human *S. suis* 2 infections have occurred occasionally worldwide [8,9]. In 1998 and 2005, two outbreaks of *S. suis* 2 in China caused severe streptococcal toxic shock-like syndrome in infected patients with mortalities as high as 62.7% to 81.3% [10]. Over the past 20 years, a growing number of studies involving *S. suis* pathogenic mechanisms have identified over 70 bacterial virulence-associated factors using comparative genomics, transcriptomics, proteomics, suppression subtractive hybridization, and other methods [11].

Many surface proteins were reported to contribute to bacterial adhesion to cells, blood invasion, immune evasion, and transmembrane nutrient delivery, such as enolase [12,13], glutamate dehydrogenase (GDH) [14], elongation factor Tu (EF-Tu) [15], sortases [16], glyceraldehyde-3-phosphate dehydrogenase (GAPDH) [17], factor H-combining protein (Fhb) [18,19], β-galactosidase (BgaC) [20], laminin-binding protein (Lmb) [21], and eukaryotic-like Ser/Thr protein kinase (STK) [22]. Recently, a family of surface-exposed proteins containing multiple histidine triad (His-x-x-His-x-His) motifs was reported to be in the *Streptococcus* genus and has attracted widespread attention [23]. The histidine triad proteins (Htps) were initially identified from *Streptococcus pneumoniae* and named pneumococcal histidine triad proteins (Phts) [24–26]. Subsequently, the homologs of Pht proteins were also identified in *Streptococcus pyogenes* (Slr and HtpA) [27,28], *Streptococcus agalacticae* (Blr and Sht) [29,30], and *Streptococcus suis* (HtpS) [31]. Based on phylogenetic relationship and domain structure analyses, *htp*s were classified into type I and type II subfamilies [32]. Our previously study found three Htps in *S. suis* 2, HtpsA (HtpS, *SSU05_0332*), HtpsB (*SSU05*_*1267*), and HtpsC (*SSU05_1577*) [31,32]. Among them, HtpsA belongs to the HTP I type subfamilies, whereas HtpsB and HtpsC belong to the HTP II type subfamilies [32]. The *S. suis* HtpsA is orthologous to the HtpA of *S. pyogenes* and PhtD of *S. pneumoniae*, which form an operon with an upstream laminin-binding protein (*lmb*)-encoding gene and is regulated by the AdcR protein [24,27,33,34]. Many studies have shown that *S. pneumoniae* PhtD is involved in a diverse range of important biological functions, including zinc-ion homeostasis, immune evasion, adherence of bacteria to host cells, and pathogenicity [23].

To elucidate the biological functions of HtpsA and its potential role in the pathogenicity of *S. suis* 2, we constructed a gene knockout mutant, Δ*htps*A, of the *S. suis* 2 strain by homologous recombination. Comprehensive experimental studies showed obvious morphology change and attenuation of pathogenicity in the mutant strain. RNA sequencing (RNA-seq) analysis suggested that the inactivation of *htps*A resulted in the downregulation of many genes involved in glucose metabolism and virulence-related factors.

## Materials and methods

### Bacterial strains, plasmids, and culture conditions

The bacterial strains and plasmids used in this study are listed in Table 1. The *S. suis* 2 virulent strain 05ZYH33 (wild-type, WT) was isolated from an infected patient during the 2005 outbreak in Sichuan, China [10]. The 05ZYH33 strain and isogenic mutant strains were grown in Todd-Hewitt broth (THB; Difco Laboratories, Detroit, MI, USA) liquid medium or plated on THB agar plates at 37°C in a 5% CO_2_ atmosphere supplemented with 5% (v/v) sheep blood when needed. Spectinomycin (Spc, Sigma, St. Louis., MO, USA) was added to screen for the *htps*A mutant strain, 100 µg/mL. The *E. coli* DH5α strain used in the construction of the recombinant gene knockout plasmid pUC::*htps*A (consisting of a spectinomycin resistance (*Spc*^R^) cassette with flanking homology regions of the *htps*A gene) was purchased from Transgen Co. (Beijing, China) and maintained in Luria-Bertani (LB) broth liquid medium or plated on LB agar at 37°C. The following antibiotics were added to the medium at the indicated concentrations: for the isogenic mutant strains, spectinomycin at 100 μg/mL; for *E. coli*, ampicillin or kanamycin [Amp, Sigma, St. Louis, MO, USA] at 100 μg/mL.

**Table 1.** Bacterial strains and plasmids used in this study.

Strains/plasmids	Characteristics and/or function	Source
**Strains**		
*S. suis* 05ZYH33	Virulent strain isolated from a patient with STSS	Lab collection
*S. suis* Δ*cps*2B	Isogenic Δ*cps*2B deletion mutant of strain 05ZYH33; *Spc^R^*	44
*S. suis* Δ*htps*C	Isogenic Δ*htps*C deletion mutant of strain 05ZYH33; *Spc^R^*	39
*S. suis* Δ*htps*A	Isogenic Δ*htps*A deletion mutant of strain 05ZYH33; *Spc^R^*	This study
*E. coli* DH5*α*	Cloning host recombinant plasmids	Transgen
**Plasmids**		
pMD18 – T	Cloning vector; *Amp^R^*	Takara
pUC19	*E. coli* cloning vector, lacZ, *Amp^R^*	Takara
pUC::*htps*A	A recombinant vector with the background of pUC19, designed for knock-out of *htps*A; *Amp^R^, Spc^R^*	This study
pSET2	*E. coli-S. suis* shuttle vector; *Spc^R^*	35

The *Amp^R^* and *Spc^R^* represent ampicillin resistant gene and spectinomycin resistant gene respectively.

### *Construction of an* htps*A knockout mutant*

Homologous recombination was utilized to generate an *htps*A mutant as described previously 22]. Primers used in this study are listed in Table S1. Using primers LA1/LA2 and RA1/RA2, the 5ʹ upstream region (976 bp) and 3ʹ fragment (847 bp) of *htps*A gene were amplified by PCR from the genome of *S. suis* 2 05ZYH33, respectively. The *Spc^R^* gene was amplified from the pSET2 plasmid using primers Spc1/Spc2. The three fragments were double-digested by restriction enzymes and then ligated into pUC19 to form the knock-out plasmid pUC::*htps*A. Then, the pUC::*htps*A plasmids were used for the electronic transformation of the 05ZYH33 competent cells. The putative transformants were confirmed by combined PCR and then verified by RT-PCR and direct DNA sequencing using a series of specific primers (Table S1).

### RNA-seq and quantitative real-time RT-PCR (qRT-PCR)

The 05ZYH33 and ∆*htps*A strains were cultured to the middle of the logarithmic phase (OD_600_ = 0.6). Bacterial cells were harvested by centrifugation at 12, 000 × g for 2 minutes. Then, 3 mg/mL of fresh lysozyme (Sigma, St. Louis, MO, USA) was used to resuspend the collected cells and incubated for 5–10 minutes. Total RNA was extracted from the 05ZYH33 and ∆*htps*A strains using an SV Total RNA Isolation System kit (Promega, Madison, Wisconsin, USA), according to the manufacturer’s instructions. The extracted RNA concentration and integrity were assessed by One Drop spectrophotometer (Pharmacia, Dübendorf, Switzerland) and electrophoresis on a 1.5% agarose gel, respectively. The RNA concentration of the 05ZYH33 and ∆*htps*A strains were determined as 551.29 ng/µL and 539.77 ng/µL, respectively. The RNA samples were stored at −80°C until needed.

The RNA samples were sent to the GENEWIZ Company (Suzhou, China) for RNA-seq. The sequencing library was performed as reported previously [36]. The amplified library was sequenced using an Illumina HiSeq^TM^ 2500 according to the manufacturer’s protocol. Transcriptome data were analyzed as follows: i) the RPKM (reads per kilobase per million mapped reads) values were calculated for each gene using uniquely mapped reads; ii) differential expression genes were confirmed via the model |log2 (fold_change)| ≥1 and *P*-value ≤0.05. The categorization of biological processes was analyzed using the Gene Ontology (GO) project (http://www.geneontology.or).

To confirm the differentially expressed genes of RNA-seq, quantitative real-time RT-PCR was performed using primers listed in table S1 as follwing: first-strand cDNA was synthesized according to the PrimeScript^TM^ RT Master Mix (Perfect Real Time) Kit (TaKaRa, Dalian, China). The cDNA samples were used for real-time RT-PCR (Applied Biosystems QuantStudio 3 Real-time PCR System, ThermoFisher, Shanghai, China). The relative levels of target gene expression were normalized with the *gapdh* gene using the 2^− ∆∆Ct^ method.

### Detection of the genetic stability of the mutant strains and growth curves

The mutant Δ*htps*A was passaged more than 50 consecutive times in THB liquid containing spectinomycin (100 μg/mL) or no spectinomycin at 37°C. The genetic stability of Δ*htps*A was assessed by PCR using the primers Spc1/Spc2. The Δ*htps*A and 05ZYH33 strains were inoculated in Colombia sheep agar plates (bioMérieux, Shanghai, China) at 37°C for 48 hours. The single colonies were inoculated into fresh THB liquid medium. Cultures were then inoculated into fresh THB at 1:100 ratios for growth curve analysis. Subsequently, the absorbance of the cultures was monitored at 1 hour intervals using a spectrophotometer (Bio-Rad, Hercules, California, USA) at a wavelength of 600 nm, and sterile THB media was used as the blank control.

### Morphological observation of the 05ZYH33 and mutant strain

The Δ*htps*A and 05ZYH33 strains were inoculated into THB liquid medium (containing 10% fetal bovine serum) and cultured to the middle of the logarithmic growth phase (OD_600_ = 0.6) at 37°C, and then washed twice with ddH_2_O. Each sample was fixed on glass slides by flaming. Gram staining was conducted according to the instructions provided in the Gram staining kit (Jian Cheng, Nanjing, China). The morphology of bacteria was observed under a light microscope (10 × 100 times).

Transmission electron microscope (TEM) observation was performed as previously described [22]. Briefly, a single colony (05ZYH33, Δ*htps*C, Δ*htps*A, or Δ*cps*2B) was picked from Colombia sheep agar plates and cultured in THB liquid medium adding 10% fetal bovine serum. The bacterial cells were harvested at OD_600_ = 0.8 and fixed in 2.5% glutaraldehyde (Sangon Biotech, Shanghai, China) for 2 hours, followed by washing with 0.01 M PBS. After that, bacterial cells were fixed in cacodylate buffer with 1% osmium tetroxide (Sigma, St. Louis, MO, USA) for 2 hours at 25°C in the dark. The cells were then dehydrated for 20 minutes with gradient acetone and then embedded in Epon-618 epoxy resin (BOC, New York, USA). Ultra-thin sections were post-stained with aqueous uranyl acetate and lead citrate and observed using a JEM-1010 TEM (JEOL, Ltd., Tokyo, Japan) at an accelerating voltage of 100 kV. The thickness of the bacterial capsule was determined by imageJ software by randomly selecting 30 cells.

### The bacterial agglutination test

A single colony of both the 05ZYH33 and Δ*htps*A strains was picked from Colombia sheep agar plates and cultured in THB liquid medium at 37°C 12 hours. Equal amounts of each sample (5 μL) were evenly coated on two glass slides. One was dripped with 5 μL of specific *S. suis* 2 antiserum (Statens Serum Institut, Copenhagen, Denmark), and the other was a negative control. The results were observed under a microscope.

### Determination of sialic acid content

Determination of sialic acid content was performed as described previously [37], with slight modifications. Briefly, the single colonies (05ZYH33 and Δ*htps*A) were picked from Colombia sheep agar plates, inoculated into THB liquid medium (containing 10% fetal bovine serum), and cultured to OD_600_ = 0.3 at 37°C. The pellets were harvested by centrifugation at 8, 000 × g for 5 minutes. After washing with PBS, the resuspended pellet was centrifuged again at 8, 000 × g for 5 minutes and resuspended in 600 μL of PBS, of which 500 μL of the suspensions was disrupted by sonication. The supernatant was collected by centrifugation at 15, 000 × g for 15 minutes at 4°C. After adding 10 μL of sialidase (0.25 U), the supernatants were hydrolyzed for 1 hour at 37°C. The sialic acid concentration was detected using a Sialic Acid Assay Kit (Jian Cheng, Nanjing, China), according to the manufacturer’s instructions. The following formula was used for calculating the sialic acid concentration:

$$\begin{array}{rl} & SA_{content}(mg/10^{10}cfu)=\frac{OD_{sample}-OD_{blank}}{OD_{standard}-OD_{blank}}\\ & \times C_{standard}\times V_{standard}\times MW_{SA}\times \frac{10^{10}}{cfu_{sample}} \end{array}$$

Note: SA represents sialic acid; OD represents optical density value at 560 nm wavelength; C represents concentration of standard mmol/L; V represents volume of standard; MW represents molecular weight of sialic acid, 309.3 g/mol.

### Adhesion assay and anti-phagocytosis assay

The bacterial strains were inoculated into THB liquid medium (containing 10% fetal bovine serum) and cultured to log phase at 37°C. Bacterial cells were collected by centrifugation at 5, 000 × rpm for 10 minutes, washed three times with PBS (pH = 7.4), and then opsonized to a density of 5 × 10^8^ CFU/mL. 5(6)-carboxyfluorescein diacetate, succinimidyl ester (CFDA-SE) was added to the resuspension at a final concentration of 10 μM and then incubated at 37°C for 30 minutes. The pellets were collected again by centrifugation at 5, 000 × rpm for 10 minutes and then washed twice with PBS. Labeled bacterial pellets were put on ice until use.

The adherence assay was performed on HEp-2 cells (ATCC CCL23) according to previous report [38]. The anti-phagocytosis ability was evaluated using Raw264.7 murine macrophages, as previously described [22]. Briefly, 1 × 10^6^ cells (HEp-2 or RAW264.7) were incubated with 1 × 10^8^ CFU of CFSE-labeled bacterial pellets at a ratio of 100:1 at 37°C for 2 hours. Additionally, for anti-phagocytosis, the cells were treated with penicillin (5 μg/mL) and gentamycin (100 μg/mL) (Sigma, St. Louis, MO, USA) for 1 hour in the dark to eliminate extracellular bacteria. After incubation, the pellets were washed three times with PBS, and then 4% (wt/vol) paraformaldehyde was added and softly mixed to fix cells. Flow cytometry was performed with a FACSCalibur (BD, Franklin Lakes, New Jersey, USA). The adhesion rates and the phagocytosis rates were assessed based on the normalized mean fluorescence intensities (NMFI).

### *The survival of 05ZYH33 and Δ*htps*A strains in human whole blood*

A whole-blood bactericidal activity was performed as previously reported [39]. The Δ*htps*A and 05ZYH33 strains were grown to the middle of the logarithmic phase (OD_600_ = 0.6) in THB liquid medium at 37°C. The pellets were then collected by centrifugation at 8, 000 × g for 10 minutes and washed twice in sterile 0.01 M PBS. The bacterial was opsonized to a density of 5 × 10^8^ CFU/mL. Then, 10 μL of the bacterial suspensions was added to 350 μL human anticoagulant whole blood and incubated in 5% CO_2_ at 37°C for 8 hours. Mixtures (50 μL) of each group sample were diluted, and the number of viable bacteria was determined by plating serial dilutions onto THB agar and incubating overnight at 37°C. The survival rate was calculated as CFU per mL. Experiments were performed in triplicate.

### Experimental infection of mice

To compare the pathogenicity of the Δ*htps*A and 05ZYH33 strains, a bacterial challenge experiment was performed in a previously constructed mouse model [22]. Four-week-old specific pathogen free (SPF) grade female BALB/c mice (SLAC, Nanjing, China) were randomly divided into three groups consisting of 10 mice and infected with 1 × 10^8^ CFU of either Δ*htpsA* or 05ZYH33 strains via intraperitoneal injection. Negative controls were treated with equal volumes of aseptic THB medium. Mice were monitored in terms of clinical symptoms for 2 weeks, and the dead mice were recorded. A Kaplan-Meier survival curve analysis was performed using the SPSS package to test the significant difference among different groups. All animal experiments were carried out according to the recommendations of the laboratory animal administration rules, State Scientific and Technological Commission. The protocol was approved by the Ethics Committee of Hua Dong Research Institute for Medicine and Biotechnics.

### Statistical analysis

Statistics for the survival curves of mice were done with the Kaplan-Meier survival analysis. The statistical analyses of capsular thickness, sialic acid content, bacterial adherence capability, survival level in human whole blood, anti-phagocytotic ability, and qRT-PCR data were carried out by using independent-samples *t* test (two-tail). All experiments were performed at least three times, and data are displayed as the mean ± SD. A *p*-value <0.05 is considered significant. In the figures, * represents *p* < 0.05, ** represents *p* < 0.01, and NS means no significant difference.

## Results

### *Construction of an* htps*A mutant*

**Figure 1.**
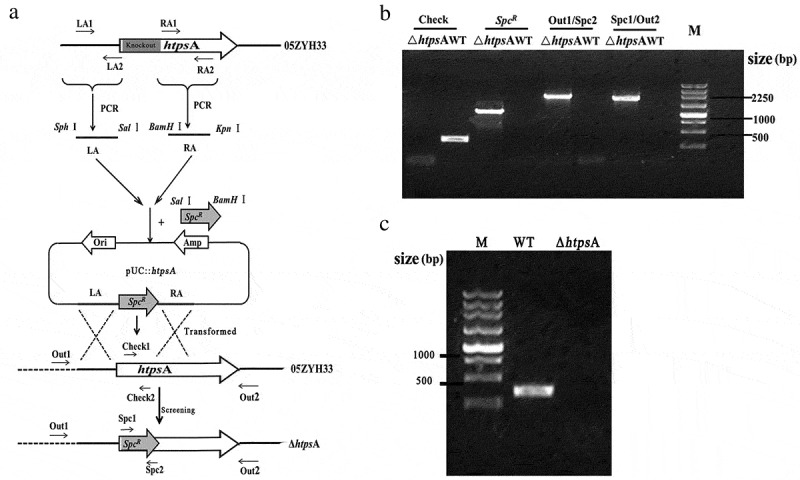
Construction of an isogenic *htps*A mutant of *S. suis* 05ZYH33. (a) Schematic diagram of the construction process of the Δ*htps*A strain. (b) Combined PCRs of the Δ*htps*A mutant. (c) Reverse-transcription PCR analysis of *htps*A gene transcripts. The primer pairs and templates used in the PCR analysis are indicated above the lanes. WT and Δ*htps*A represent genome DNA of the wild-type strain 05ZYH33 and mutant strain, respectively.

An isogenic mutant strain, Δ*htps*A, was constructed through homologous recombination as illustrated in Figure 1a. Using the primers of Check1/Check2, two *htps*A negative strains were identified by PCR amplification after screening more than 100 *Spc*^R^ transformants. Combined PCR analysis showed that the 5ʹ 970-bp region of *htps*A gene was successfully replaced by the *Spc*^R^ gene in the mutant strain (Figure 1b). RT-PCR also confirmed the lack of expression of *htps*A from the mutant strain (Figure 1c). Finally, direct DNA sequencing showed the 5ʹ 970-bp region of *htps*A gene was completely displaced by the *Spc*^R^ gene in the mutant (data not show). Taken together, an *htps*A mutant, named Δ*htps*A, was successfully constructed and used for subsequent experiments in this study.

### *Morphological alteration of the Δ*htps*A*

**Figure 2.**
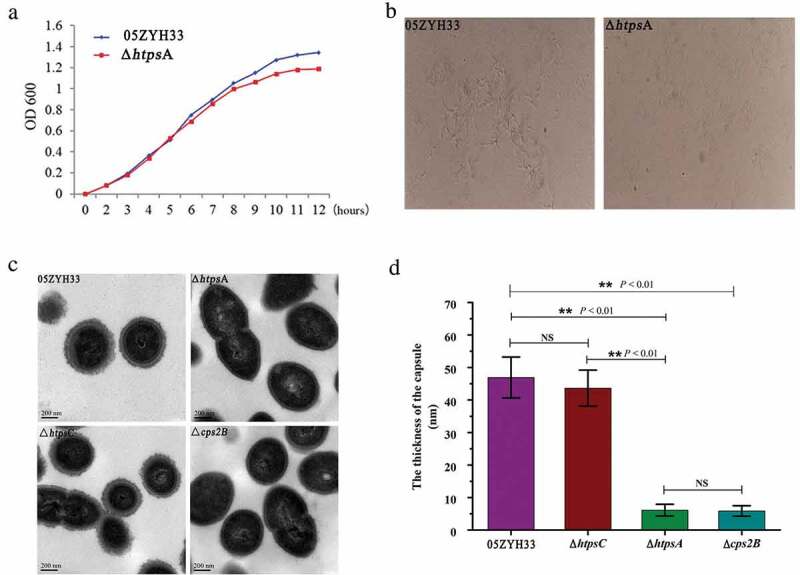
Phenotypic analysis of the 05ZYH33 and Δ*htps*A mutant strains. (a) Cell density was measured spectrophotometrically at a wavelength of 600 nm. (b) Antiserum aggregation reaction of the Δ*htps*A and 05ZYH33. (c) Observation of capsular morphology of the Δ*htps*A, Δ*htps*C, Δ*cps*2B and the wild-type strains by transmission electron microscopy. (d) Determination and analysis of capsule thickness of the Δ*htps*A, Δ*htps*C, Δ*cps*2B and wild-type strains. ** represents significant differences (*P* < 0.01), NS represents no significant differences.

To investigate whether inactivation of the *htps*A gene affected the morphology of *S. suis* 2, several features were assessed and compared between the Δ*htps*A and 05ZYH33 strains, including growth rates, bacterial shape, capsule formation, and colony morphology. Under normal growth conditions without antibiotics, the growth density of Δ*htps*A was lower than the 05ZYH33 strain starting from the late logarithmic stage (7 hours), and the difference increased after entering the stationary stage (Figure 2a). There were no significant differences between the two groups regarding colony morphology, including size, color, shape, transparency, and stickiness when growing on the solid medium. Transmission electron microscopy (TEM) observation revealed that the capsular structure of Δ*htps*A was obviously thinner than that of the 05ZYH33 (Figure 2c). The capsular structure of Δ*htps*A is similar to a capsular deficiency strain by *cps*2B mutation. However, similar phenotype of capsular deficiency was not observed in the *htps*C mutant strain (Figure 2c). We calculated the capsular thickness of the 05ZYH33, Δ*htps*A, Δ*htps*C and Δ*cps*2B strain, and found that Δ*htps*A cells (6.11 ± 1.81 nm) were significantly thinner than that of 05ZYH33 (46.92 ± 6.30 nm) and Δ*htps*C (43.64 ± 5.55 nm), as shown in Figure 2d.

We further performed an agglutination test using a specific antibody against the capsule of *S. suis* 2 in order to confirm the deficiency in capsular development of the Δ*htps*A. The Δ*htps*A aggregated weakly 3 minutes after adding the specific antibody, while apparent cell aggregation for the 05ZYH33 strain was observed at 1 minute after the treatment (Figure 2b). These results suggested that the insensitivity of the Δ*htps*A to specific antiserum may be related to the loss of capsular structure.

### *Reduced sialic acid production in the Δ*htps*A*

**Figure 3.**
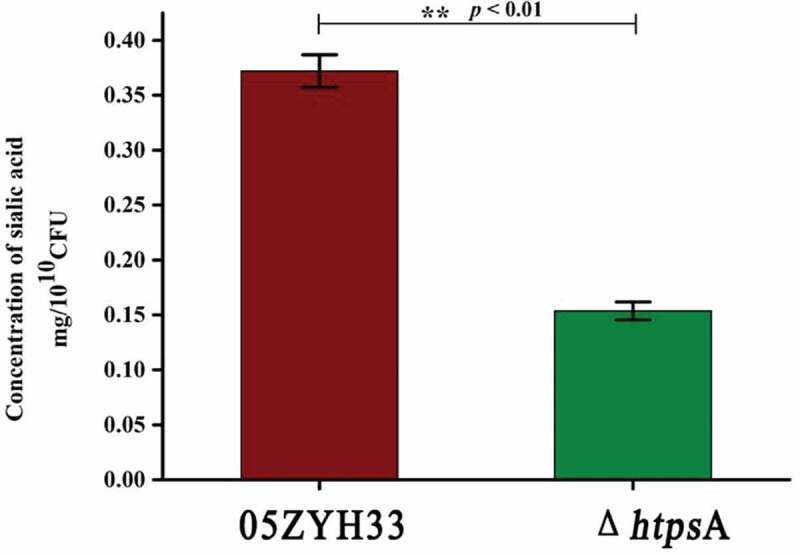
Effects of *htps*A deletion on bacterial capsular sialic acid content. Determination of sialic acid content of the Δ*htps*A and the wild strain 05ZYH3. (** indicates *P < *0.01, Student’s *t-test*).

Sialic acid is an important component of the capsule of *S. suis* 2. Inactivation of the sialic acid synthesis pathway resulted in the loss of the capsular structure. In order to examine whether the observed capsule-less structure of the Δ*htps*A strain was associated with the lack of sialic acid, we detected the sialic acid content of the Δ*htps*A strain. Our results showed that the content of sialic acid in the capsule of the Δ*htps*A cells was only half of the sialic acid content in 05ZYH33 cells (Figure 3).

### *Attenuated pathogenicity of the Δ*htps*A*

**Figure 4.**
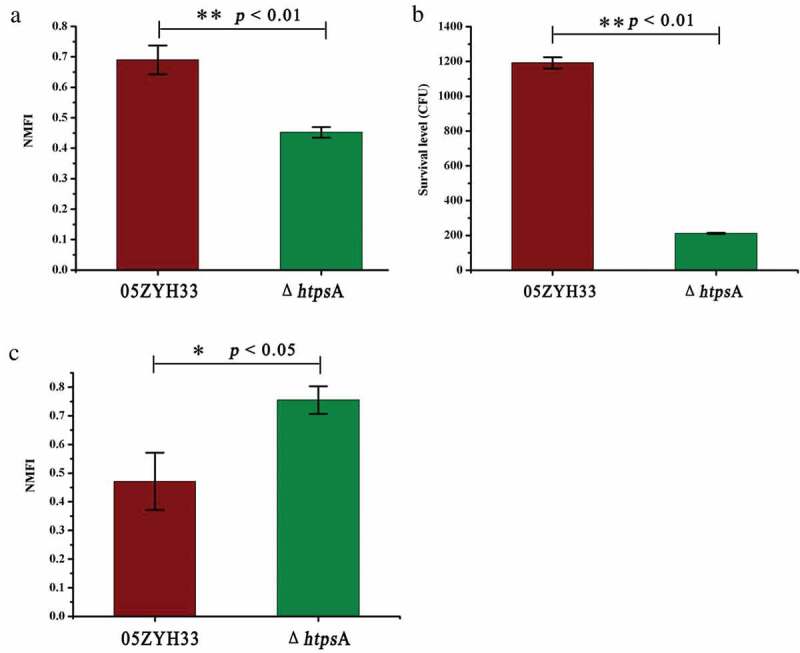
Effect of HtpsA deficiency on virulence and pathogenicity of bacteria. (a) Comparison of bacterial adherence capability of the Δ*htps*A mutant with the wild-type 05ZYH33 strain. The normalized mean fluorescence intensities (NMFI) of HEp-2 cells after incubation with the bacteria are shown as columns with standard errors. (b) Survival of the 05ZYH33 and Δ*htpsA* mutant in human whole blood. Mixtures were incubated at 37°C for 8 hours, and then the dilutions were coated on agar plates. The number of single colonies that grew after incubating overnight was counted. (c) Evaluation of the anti-phagocytotic ability of *S. suis* strains in macrophage RAW264.7 cells (* indicates *P < 0.05*; ** indicates *P < 0.01*, Student’s *t-test*).

To evaluate the adhesive capacity of the Δ*htps*A strain, CFSE-labeled Δ*htps*A and 05ZYH33 cells were co-cultured with HEp-2 cells. Our results showed that the NMFI value of HEp-2 cells incubated with the Δ*htps*A declined nearly 40% as compared to the 05ZYH33 strain (*P* < 0.01, Figure 4a). This result indicated that the inactivation of *htps*A significantly impaired the adherence of *S. suis* 2.

Subsequently, a whole-blood killing test showed that the survival rate of the Δ*htps*A strain dropped sharply from 1188 CFU to 213 CFU as compared with the 05ZYH33 strain (*P* < 0.05, Figure 4b). We further compared the anti-phagocytosis ability of the Δ*htps*A and 05ZYH33 strains using murine RAW264.7 macrophages. As shown in Figure 4c, the NMFI value of the mutant strain was 0.75 ± 0.047, which was approximately a 1.27-fold increase as compared with the 05ZYH33 strain (0.47 ± 0.099). This indicated that the sensitivity of Δ*htps*A to phagocytic cells increased as compared to the 05ZYH33 strain, which resulted in a significantly weakened anti-phagocytosis ability in the Δ*htps*A (*P* < 0.05). All these results suggested that the deletion of *htps*A attenuated the pathogenicity of *S. suis* 2 via different aspects.

### *Weakened virulence of the* htps*A mutant in the mouse infection model*

**Figure 5.**
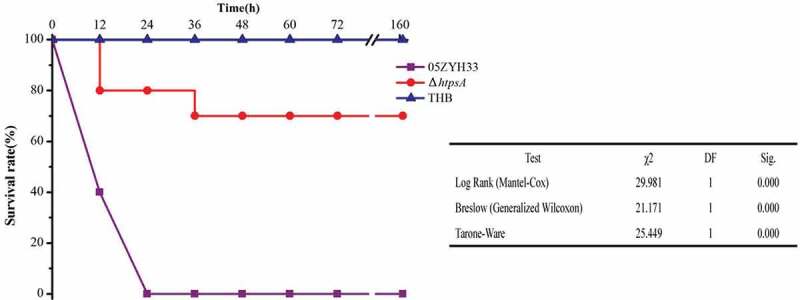
Survival curves of mice infected with the Δ*htps*A mutant or wild-type strain 05ZYH33 strains. Four-week-old BALB/c mice were challenged intraperitoneally with 1 × 10^8^ CFU bacteria, and the survival time was monitored. * represents a significant difference of *P* < 0.05, and ** represents a significant difference of *P* < 0.01.

A mouse infection assay was used to clarify the contribution of the *htps*A gene to bacterial virulence. The mice were randomly divided into three groups, namely Δ*htps*A, 05ZYH33, and THB groups as negative control. After 12 hours of infection, the survival rate of the Δ*htps*A group was 80%, while the 05ZYH33 group was only 40%. Moreover, after infection 24 hours, the survival rate was still 80% in the Δ*htps*A group, while all the mice in the 05ZYH33-infected group died. The survival rate was 70% in the Δ*htps*A group at 36 hours after infection (Figure 5). No symptoms were found in the surviving mice through the end of the 7-day experiment. In the THB group, all mice were in good condition. Statistical analysis revealed that the rate of mortality was significantly reduced in the Δ*htps*A group (*P* < 0.01, Kaplan-Meier survival analysis). The results of animal experiments demonstrated that knockout of the *htps*A gene impaired the full virulence of 05ZYH33.

### *Altered transcription profiles of the* htps*A mutant*

To explore the mechanisms underlying the function of *htps*A, the transcriptome profile of both the Δ*htps*A and 05ZYH33 strain was determined by RNA-seq. Compared with the 05ZYH33 strain, there were 98 genes downregulated and 28 genes upregulated in the Δ*htps*A, among which five were located on the 89 K pathogenic island (Table S2). The differentially expressed genes were classified into different functional categories (Table 2), including physiological metabolism (45.2%), enzymes associated with transport systems (21.4%), genetic information processing (10.3%), and function-unknown genes (21.4%). Notably, 51% (50 of 98 genes) of the downregulated genes encoded proteins involving in saccharometabolism and sugar transporters, such as GalK, GalT, GlgC, and MalM (Table 3). This may be one of the most important reasons for the reduction in the bacterial capsular structure in the Δ*htps*A strain. The expression of several virulence-related factors was also observed, including suilysin and genes encoding hyaluronidase (Table S2).

**Table 2.** Functional classification of differentially expressed genes between the Δ*htps*A mutant and 05ZYH33 strain.

Functional classification	Downregulated		Upregulated
**1. Metabolism**	47	10	
1.1 Carbohydrate	32	0	
1.2 Lipid	2	6	
1.3 Nucleotide	4	1	
1.4 Amino acid	4	3	
1.5 Energy production	5	0	
**2. Transport systems**	25	2	
**3. Genetic Information Processing**	13	0	
3.1 Information storage and processing	5	0	
3.2 Cellular processes and signaling	4	0	
3.3 Protein biosynthesis	4	2	
**4. Unknown or hypothetical proteins**	13	14	
**Total**	98	28

**Table 3.** Classification of downregulated genes related to saccharometabolism.

Functional classification	Number of variable genes	Annotation of key genes
Glucose metabolism-related enzymes	13	*glgC, glpK, malM, uxaC*, endo-beta-N-acetylglucosaminidase
Galactose metabolism-related enzymes	7	*galK, galt, galM*, beta-galactosidase, alpha-galactosidase
Mannose metabolism-related enzymes	3	N-acetylmannosamine 6-P epimerase, putative alpha-1,2-mannosidase
Other sugar metabolism-related enzymes	5	*gtfA*, beta-fructosidases, beta-hexosamidase, sugar kinases
PTS system	9	PTS-EIIB; PTS-EIID; PTS-EIIC
ABC-type sugar transport system	13	*msmE, malX, malC*, ABC-type sugar transport systems, permease components

**Figure 6.**
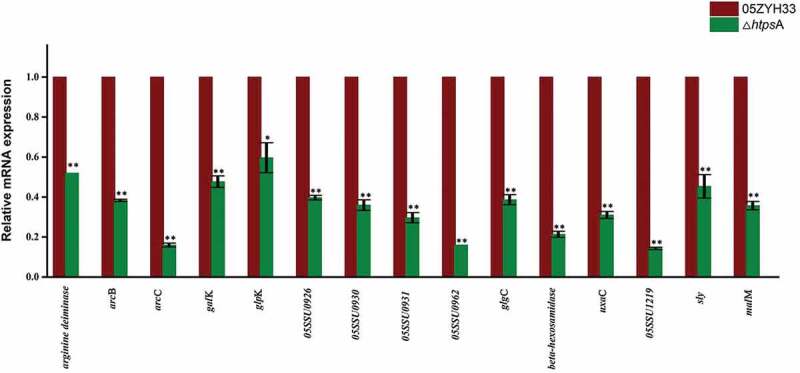
qRT-PCR validation of expression profiles of 15 differentially expressed genes identified by RNA-seq. The house-keeping gene *gapdh* was used as internal control, and error bars represent SEM of three replicates. * represents a significant difference of *P* < 0.05, and ** represents a significant difference of *P* < 0.01. Significant difference as determined by *Student*’s *t*-test.

Fifteen genes from different functional catagories were selected for further qRT-PCR analysis to confirm their differential expression after *htps*A inactivation. As shown in Figure 6, the results obtained from the qRT-PCR analysis are highly consistent with those in the RNA-seq data. Specifically, the expression of several proved or potential virulence factor-related genes including *sly, arcB, arcC* and *arginine deiminase* was downregulated 2.22, 2.61, 6.28 and 1.93 folds compared to the 05ZYH33 strain, respectively. The expression level of four genes (*05SSU0926, 05SSU0930, 05SSU0931, 05SSU0962*) from 89 K virulence island was also decreased by two to six folds. The expression of genes associated with glycometabolism including *malM, uxaC, galK, beta-hexosamidase* and *05SSU1219* (PTS system, mannose specific) were downregulated 2.8, 3.21, 2.11, 4.68 and 7.04 folds, respectively. Taken together, our data revealed that inactivation of *htps*A influenced expression of multiple genes involved in metabolism processes, capsule synthesis and virulence of the bacterium. We also evaluated the expression of the other two *htp* family members *htps*B and *htps*C by qRT-PCR. No significant differential expression was observed for the two genes as revealed by the RNA-seq data (Figure S1).

## Discussion

Streptococci express many surface proteins to promote host infection [40]. *htps* are a group of genes widely distributed within the *Streptococcus* genus and play important roles in bacterial infection [23]. According to the phylogenetic relationship and gene structure, *htps* can be divided into two subfamilies: HTP I and HTP II [32]. Research regarding four HTP I subfamily members, *phtA, phtB, phtD*, and *phtE*, in *S. pneumoniae* found that the virulence of the bacteria was weakened or even lost when deleting two or more members at the same time, but there was no effect on bacterial virulence when only one gene was inactivated [34]. This report suggested that the *pht* family proteins are very important for the pathogenesis of *S. pneumonia*, although functional redundancy existed between family members. Furthermore, deletion of *pht*D orthologous genes resulted in decreased virulence of *S. agalactiae* and *S. pyogenes*, indicating that the family proteins are closely related to the pathogenesis [27,30]. Recently, our study showed that a type II Htp protein, HtpsC, of *S. suis* 2 could bind to laminin and fibronectin of the human ECM complex. Inactivation of *htps*C significantly affected adherence and attenuated the virulence of *S. suis* 2 in mice [39]. In this study, we found that the deletion of the type I Htp member, *htps*A, in *S. suis* 2, impaired the full virulence and the capsular structure of the bacterium. The results from our present and previous studies suggested that type I and type II proteins play important, but not redundant, roles in the virulence of *S. suis* 2.

### *The deficiency in capsular development is one of the reasons for the weakened virulence in the Δ*htps*A strain*

Capsular polysaccharide (CPS) is an important virulence factor for several pathogens. It is involved in many infection processes in *S. suis*, such as bacterial adhesion, invasion, survival, and blocking neutrophil and monocyte/macrophage-mediated phagocytosis and killing [41]. CPS biosynthesis involves 25 open reading frames in *S. suis* 2, including *orf2Z, orf2Y, orf2X, orf2L, orf2M, orf2N, orf2U, orf2V, Cps2A-Cps2K*, and *Cps2O-Cps2T* [42]. An unencapsulated *S. suis* 2 mutant generated by inactivating the *cps2* gene exhibits a 15–60% decrease in the adherence to HEp-2 cells [43]. And inactivation of *cps*2B caused a significant loss of the capsule and decreased the pathogenicity of *S. suis* 2 in a mouse infection model [44]. The deletion of four genes (*cps2E, cps2 G, cps2 J*, and *cps2 L*) in *S. suis* 2 SC19 caused a significant decrease in the capsular sialic acid synthesis and virulence [45]. In this study, we observed that the Δ*htps*A had a thinner capsule than 05ZYH33, which likely caused an increase in the sensitivity of this strain to phagocytosis by macrophages. The reduced content of sialic acid, one of the main components of the capsular polysaccharide, in the Δ*htps*A also supports its deficiency in capsular development. Furthermore, our RNA-seq analysis of the Δ*htps*A strain revealed that among the 98 downregulated genes, 51% are involved in the metabolism of glucose, galactose, mannose, PTS, and ABC-type sugar transport systems (Table 3). A possible hypothesis is that the absence of *htps*A caused a dysfunction in glucose metabolism and glucose transport system in the Δ*htps*A, which resulted in the hindrance of bacterial capsular polysaccharide synthesis and loss of the typical capsular structure. Notably, capsular deficiency was not detected for the *htps*C mutant strain, suggesting functional divergence of the two *htp* family members in *S. suis* 2 morphology development. Together, these resulted in the decreased anti-phagocytosis ability and attenuated virulence of the Δ*htps*A in a murine infection model.

### *The downregulated expression of virulence-related factors may also contribute to the weakened virulence of the Δ*htps*A* *strain*

Virulence factors play important roles in different stages of pathogen infection and pathogenicity [40]. Among numerous virulence factors, *suilysin* (*SLY*), a vital virulence factor, has been verified to participate in the bacterial infection process through activating phagocytes and inducing the release of proinflammatory cytokines [46]. In this study, the *SLY* gene was downregulated 2.22-fold in the *htps*A mutant strain as compared with the 05ZYH33 strain. We also observed the decreased expression of arginine deiminase (*arc*A, *SSU05_0624*), ornithine carbamoyltransferase (*arc*B, *SSU05_0626*), and carbamate kinase (*arcC, SSU05_0627*) in the *htps*A mutant strain. The ArcA is a member of arginine deiminase system (ADS), which is a secondary metabolic system that exists in many different bacterial pathogens and is often associated with virulence [47]. In *S. pyogenes*, the ADS is involved in adhesion and invasion of epithelial cells [48]. In *S. suis*, ADS is responsible for survival under acidic conditions, where it catalyzes the conversion of arginine to ornithine, ammonia, and carbon dioxide [49]. Additionally, the genes encoding hyaluronidase (*SSU05_1212* ~ *SSU05*_*1215*), which catalyzes the degradation of hyaluronic acid (HA), were downregulated 3.25 to 3.62-fold in the Δ*htps*A strain. In *Streptococcus* and *Staphylococcus*, hyaluronidases are virulence factors that contribute to the destruction of the polysaccharide in the basement layer to facilitate dissemination through the tissues of the host organism [50]. Hyaluronidase activity enables GBS to subvert uterine immune responses, leading to increased rates of ascending infection and preterm birth [51]. Hyaluronate lyase may be also a potential virulence factor in *S. suis*, which requires hyaluronic acid as a carbon source [52].

Sugar Phosphotransferase System (PTS) mediates the uptake and phosphorylation of carbohydrates and controls the carbon and nitrogen metabolism in response to the availability of sugars [53]. In *Listeria monocytogenes*, two pairs of soluble PTS components (EIIACel1/EIIBCel1 and EIIACel2/EIIBCel2) and the permease EIICCel1 were responsible for cellobiose uptake and repression of PrfA, which is a virulence gene activator [54]. As a virulence gene, the *ptsP* mutant caused a decrease in the colonization ability and pathogenicity of *Legionella pneumophila* [55]. These reports together suggested that the PTS components are closely related to virulence. There are 27 genes encoding PTS components in *S. suis* 2 05ZYH33 [56]. We noted the knockout of *htps*A resulted in a significantly downregulated expression of 11 genes. These genes may play an important role in the transport and metabolism of bacterial carbohydrates and the regulation of virulence. Additionally, the deletion of *htps*A in this study caused a significant downregulation of 15 ABC-type transporters – most of these genes were annotated as relating to sugar metabolism. Furthermore, the ABC transporter plays an important role in the pathogenesis of several pathogenic bacteria [57–59]. Taken together, it could be inferred that the downregulation of the virulence-related factors mentioned above may also contribute to the attenuated infection and pathogenicity of *S. suis* 2.

### *The disruption of zinc homeostasis may be the third reason for the weakened virulence of the Δ*htps*A strain*

The histidine triad protein is not only related to adhesion and virulence but also to the absorption of zinc ions. The crystal structure analysis of the PhtA protein fragment and the high-resolution NMR structure of PhtD form *S. pneumoniae* have indicated that the histidine triad domain is a Zn^2+^ binding domain [60,61]. Other studies from *S. pyogenes* HtpA and *S. pneumonia* PhtD confirmed that Htp proteins did have zinc ion-binding activity [27,62]. Ogunniyi *et al*. revealed that Pneumococcal Pht proteins were regulated by the Zn^2+^-dependent repressor AdcR [34]. These studies suggested that this family of proteins play an important role in maintaining the zinc ion balance in bacteria during bacterial infection. *S. suis* 2 HtpsA exhibits high amino acid similarity (57% and 46%) to HtpA of *S. pyogenes* and PhtD of *S. pneumoniae* [31]. *S. suis* AdcR protein is able to bind to the promoter region of the *hpts*A gene [63], suggesting that it plays a role in zinc homeostasis in *S. suis* 2. Zinc ions are not only a necessary nutritional requirement for the growth of all cells and the activity of a wide variety of enzymes but also play an important role in the process of sugar metabolism. A recent study demonstrated that disordered zinc balance impairs glucose metabolism through the inhibition of the glycolytic enzymes phosphofructokinase and glyceraldehyde-3-phosphate dehydrogenase, resulting in decreased capsule biosynthesis via the inhibition of phosphoglucomutase [64]. This supports the observed transcriptional alteration of glycometabolism and capsular biosynthesis-related genes in the Δ*htpsA*.

The involvement of *htps*A in multiple biological processes largely explained the phenotype alteration of the Δ*htps*A strain. As observed in this study, the survival rate of Δ*htps*A in whole blood sharply decreased comparing to the wild-type strain. This is in accordance with the results of our previous study, which showed that incubating *S. suis* 2 with anti-HtpsA antiserum could reduce its survival rate in whole blood [31]. The previous study also showed that immunization of mice with recombinant HtpsA confers significant protection against wild-type *S. suis* 2 infection [31], which mirrors the active role of HtpsA during bacterial infection. Besides the obvious morphological alteration, the Δ*htps*A also showed slight growth reduction. Although many of the observed morphological change, including capsule deficiency, low sialic acids content, decreased adherence ability and macrophage resistance would not be affected a lot by the subtle delay in growth, we could not rule out that the growth reduction may indirectly contribute to the pathogenicity attenuation of the bacteria.

In conclusion, this work demonstrated that inactivation of *htps*A disturbed a diverse of cellular activities in *S. suis* 2, including glycometabolism, nutrient transport (PTS, ABC transporter), zinc homeostasis, and virulence factor (e.g., ADS, suilysin, and hyaluronidase) expression. Among these downregulated genes, the altered expression of saccharometabolism and sugar transporters-related genes was the most obvious. These resulted in the thinning of the bacterial capsule and attenuation of pathogenicity of the Δ*htps*A strain. In summary, our findings provide evidence that *htps*A contributes to the virulence of *S. suis* 2 in a murine infection model through a complicated mechanism.

## Supplementary Material

Supplemental Material

Supplemental Material
