# Supplementary material for: Inactivation of the htpsA gene affects capsule development and pathogenicity of Streptococcus suis
Source: Virulence. 2020 Aug 20;11(1):927–40. doi: 10.1080/21505594.2020.1792080 (PMC7567435; doi:10.1080/21505594.2020.1792080)
Supplement: Supplemental Material [file KVIR_A_1792080_SM4557.docx]

Table S2. Differentially expressed genes between the Δ*htps*A and 05ZYH33 strain identified by RNA-seq.

| **Gene Codes** | **Gene** | | **Annotation** | **Fold change*** | |
| --- | --- | --- | --- | --- | --- |
| **Up-regulated** | | | | | |
| SSU05_0229 | - | LysM domain protein | | | 2.838 |
| SSU05_0273 | - | Methyl-accepting chemotaxis protein | | | 2.116 |
| SSU05_0274 | - | Methyl-accepting chemotaxis protein | | | 2.016 |
| SSU05_0509 | *-* | Hypothetical protein | | | 1.725 |
| SSU05_0635 | - | Hypothetical protein | | | 2.964 |
| SSU05_0636 | - | Hypothetical protein | | | 3.282 |
| SSU05_0691 | - | Predicted membrane protein | | | 1.933 |
| SSU05_0756 | - | Predicted membrane protein | | | 2.091 |
| SSU05_0815 | *guaC* | GMP reductase | | | 2.301 |
| SSU05_0911 | *-* | ABC transporter, NBP/MSD fusion protein | | | 1.907 |
| SSU05_0912 | - | putative asparagine synthetase | | | 1.964 |
| SSU05_1026 | *glnp* | ABC-aminoacidtransport | | | 2.260 |
| SSU05_1371 | *sao* | Sao protein | | | 2.718 |
| SSU05_1373 | *-* | Xaa-Pro aminopeptidase | | | 1.697 |
| SSU05_1659 |  | Uncharacterized protein conserved in bacteria | | | 2.001 |
| SSU05_1663 | - | Methyl-accepting chemotaxis protein | | | 2.219 |
| SSU05_1664 | - | Hypothetical protein | | | 1.997 |
| SSU05_1792 | - | Hypothetical protein | | | 2.132 |
| SSU05_1793 | - | Hypothetical protein | | | 2.194 |
| SSU05_1796 | *accA* | Acetyl-CoA carboxylase alpha subunit | | | 2.793 |
| SSU05_1799 |  | Biotin carboxylase | | | 3.121 |
| SSU05_1801 | *accB* | Biotin carboxyl carrier protein | | | 3.412 |
| SSU05_1802 | *fabF* | 3-oxoacyl-(acyl-carrier-protein) synthase | | | 3.556 |
| SSU05_1803 | *fabG* | Dehydrogenases with different specificities | | | 3.578 |
| SSU05_1806 | - | Acyl carrier protein | | | 2.201 |
| SSU05_2031 | - | Hypothetical protein | | | 2.177 |
| SSU05_2032 | - | Conserved hypothetical protein | | | 2.239 |
| SSU05_2173 | - | Lysm domain protein | | | 2.609 |
| **Down-regulated** | | | | | |
| SSU05_0026 | *-* | SAICAR synthase | | | -2.885 |
| SSU05_0027 | - | FGAM synthase | | | -2.306 |
| SSU05_0048 | - | ABC-type multidrug transport system, permease components | | | -2.630 |
| SSU05_0170 | - | ABC-type sugar transport systems, permease components | | | -2.958 |
| SSU05_0171 | - | ABC-type sugar transport system, permease component | | | -2.575 |
| SSU05_0172 | - | Alpha-galactosidase | | | -2.210 |
| SSU05_0212 | - | Phosphotransferase system cellobiose-specific component IIC | | | -3.386 |
| SSU05_0213 | - | Hypothetical protein | | | -3.673 |
| SSU05_0214 | - | ABC-type xylose transport system, periplasmic component | | | -2.214 |
| SSU05_0280 | *adhE* | NAD-dependent acetaldehyde dehydrogenases | | | -1.794 |
| SSU05_0359 |  | Transcriptional regulators | | | -2.187 |
| SSU05_0360 | *galK* | Galactokinase | | | -3.968 |
| SSU05_0361 | *galt* | Galactose-1-phosphate uridyltransferase | | | -4.564 |
| SSU05_0372 |  | Histone acetyltransferase HPA2 and related acetyltransferases | | | -1.672 |
| SSU05_0373 | - | Amidases related to nicotinamidase | | | -1.620 |
| SSU05_0389 | *clpL* | ATP-dependent protease ATP-binding subunit | | | -2.621 |
| SSU05_0439 | - | Ribosome-associated protein Y (PSrp-1) | | | -2.248 |
| SSU05_0449 | - | Beta-galactosidase | | | -4.168 |
| SSU05_0450 | - | PTS-EIIB; PTS system, mannose-specific IIB component | | | -4.525 |
| SSU05_0451 | - | PTS-EIIC; PTS system, mannose-specific IIC component | | | -4.286 |
| SSU05_0452 | - | PTS-EIID; PTS system, mannose-specific IID component | | | -4.322 |
| SSU05_0454 | *galM* | Aldose 1-epimerase | | | -1.938 |
| SSU05_0624 | - | arginine deiminase | | | -3.511 |
| SSU05_0626 | *arcB* | Ornithine carbamoyltransferase | | | -4.066 |
| SSU05_0627 | *arcC* | Carbamate kinase | | | -4.847 |
| SSU05_0628 | - | Predicted membrane protein | | | -5.172 |
| SSU05_0654 |  | Predicted membrane metal-binding protein | | | -2.355 |
| SSU05_0717 |  | Glycerol dehydrogenase and related enzymes | | | -2.774 |
| SSU05_0722 | *glpk* | Glycerol kinase | | | -2.930 |
| SSU05_0723 |  | alpha-glycerophosphate oxidase | | | -2.498 |
| SSU05_0902 | - | Predicted hydrolase (HAD superfamily) | | | -2.282 |
| SSU05_0925 | - | putative membrane protein | | | -3.459 |
| SSU05_0926 |  | hypothetical protein | | | -2.464 |
| SSU05_0930 | - | transcriptional regulator | | | -3.259 |
| SSU05_0931 | - | hypothetical protein | | | -3.077 |
| SSU05_0962 | - | SNF2 family protein | | | -1.855 |
| SSU05_1000 | - | putative 5'-nucleotidase | | | -1.738 |
| SSU05_1013 | *glgC* | ADP-glucose pyrophosphorylase | | | -1.865 |
| SSU05_1139 | - | hypothetical protein | | |  |
| SSU05_1153 | - | beta-hexosamidase | | | -2.423 |
| SSU05_1154 | - | beta-hexosamidase | | | -2.514 |
| SSU05_1155 | - | Phosphatases | | | -2.516 |
| SSU05_1156 |  | Dehydrogenases with different specificities | | | -2.531 |
| SSU05_1157 |  | D-mannonate dehydratase | | | -2.763 |
| SSU05_1158 | *uxaC* | Glucuronate isomerase | | | -2.957 |
| SSU05_1159 | *uxaC* | Glucuronate isomerase | | | -2.993 |
| SSU05_1163 | - | Beta-galactosidase/beta-glucuronidase | | | -2.277 |
| SSU05_1205 | *citB* | Aconitase A | | | -2.175 |
| SSU05_1211 |  | hypothetical protein | | | -2.777 |
| SSU05_1212 | *hysA* | hyaluronidase | | | -3.252 |
| SSU05_1213 | *hylA* | hyaluronidase | | | -3.515 |
| SSU05_1215 | - | hyaluronidase | | | -3.617 |
| SSU05_1216 | - | Preprotein translocase subunit YajC | | | -2.584 |
| SSU05_1217 | - | PTS system, mannose -specific component IID | | | -2.806 |
| SSU05_1218 | - | PTS system, mannose -specific component IIC | | | -2.971 |
| SSU05_1219 | - | PTS system, mannose -specific component IIB  system,mannosemannose/fructose/N-acetylgalactosamine-specific component IIB | | | -3.334 |
| SSU05_1220 | - | Hypothetical protein | | | -3.300 |
| SSU05_1223 | - | Sugar kinases, ribokinase family | | | -2.302 |
| SSU05_1224 | - | Ribose 5-phosphate isomerase RpiB | | | -2.594 |
| SSU05_1225 |  | Gluconate 5-dehydrogenase | | | -2.801 |
| SSU05_1337 | - | Beta-fructosidases | | | -2.095 |
| SSU05_1338 |  | ABC-type sugar transport system, periplasmic component | | | -2.319 |
| SSU05_1339 | - | ABC-type sugar transport system, permease component | | | -2.738 |
| SSU05_1340 | - | ABC-type polysaccharide transport system, permease component | | | -2.774 |
| SSU05_1387 |  | amylase-binding protein B | | | -2.347 |
| SSU05_1401 | - | Phosphotransferase system IIC components | | | -2.650 |
| SSU05_1402 | - | N-acetylmannosamine 6-P epimerase | | | -3.380 |
| SSU05_1403 | *sly* | suilysin | | | -2.633 |
| SSU05_1555 | *gtfA* | Glycosidases | | | -5.014 |
| SSU05_1556 | - | ABC-type sugar transport systems, permease components | | | -4.236 |
| SSU05_1557 | - | ABC-type sugar transport systems, permease components | | | -4.377 |
| SSU05_1558 | *msmE* | ABC-type sugar transport system, periplasmic component | | | -5.133 |
| SSU05_1560 |  | Alpha-galactosidase | | | -4.900 |
| SSU05_1817 | - | Phosphotransferase system IIC components | | | -2.846 |
| SSU05_1907 | - | ABC-type sugar transport systems, ATPase components | | | -2.608 |
| SSU05_1915 | - | ABC transporter membrane spanning permease - sugar transport | | | -2.861 |
| SSU05_1917 |  | glycosyl hydrolase-related protein | | | -2.758 |
| SSU05_1919 | - | hypothetical protein | | | -2.829 |
| SSU05_1921 | - | Putative alpha-1,2-mannosidase | | | -2.916 |
| SSU05_1922 | - | endo-beta-N-acetylglucosaminidase | | | -2.382 |
| SSU05_1931 | - | PTS system, sucrose-specific IIBC component | | | -2.887 |
| SSU05_1957 | - | Dihydroxyacetone kinase | | | -2.490 |
| SSU05_1958 | - | Dihydroxyacetone kinase | | | -2.883 |
| SSU05_1960 | - | Glycerol uptake facilitator and related permeases | | | -2.866 |
| SSU05_1982 | *prtA* | Subtilisin-like serine proteases | | | -3.290 |
| SSU05_2060 |  | 3-hexulose-6-phosphate synthase and related proteins | | | -2.773 |
| SSU05_2063 | - | Uncharacterized protein conserved in bacteria | | | -2.480 |
| SSU05_2064 | - | Type II secretory pathway, PulA and related glycosidases | | | -2.915 |
| SSU05_2065 | - | Type II secretory pathway, PulA and related glycosidases | | | -3.302 |
| SSU05_2071 | - | Phosphotransferase system cellobiose-specific component IIC | | | -2.226 |
| SSU05_2076 | - | Transcriptional antiterminator | | | -2.409 |
| SSU05_2077 | - | Uncharacterized conserved protein | | | -2.887 |
| SSU05_2131 | *malM* | 4-alpha-glucanotransferase (amylomaltase) | | | -2.628 |
| SSU05_2132 | *malM* | 4-alpha-glucanotransferase (amylomaltase) | | | -2.731 |
| SSU05_2133 | *malX* | ABC transporter substrate-binding protein | | | -2.944 |
| SSU05_2134 | *malC* | ABC-type sugar transport systems, permease components | | | -2.123 |
| SSU05_2145 | *-* | glucocerebrosidase | | | -2.477 |
| SSU05_2147 | *-* | beta-glucosidase | | | -2.368 |

Note: Fold change indicates the multiple of transcriptional change of the gene in the mutant, the positive number indicates the up-regulation, and the negative number indicates the down-regulation.
