# Supplementary material for: Inactivation of the htpsA gene affects capsule development and pathogenicity of Streptococcus suis
Source: Virulence. 2020 Aug 20;11(1):927–40. doi: 10.1080/21505594.2020.1792080 (PMC7567435; doi:10.1080/21505594.2020.1792080)
Supplement: Supplemental Material [file KVIR_A_1792080_SM4514.docx]

Table S1. Primers used for PCR amplification and detection.

| **Primers** | **Sequences (5'-3')^a^** | **Restriction enzyme** | **Amplification target** |
| --- | --- | --- | --- |
| **General PCR** |  |  |  |
| LA1 | CGGCATGCGAAAGTGATAAGAGG | *Sph* I | Upstream border of *htpsA* |
| LA2 | CGTCGACTACGGAGCCAACAACT | *Sal* I | Upstream border of *htpsA* |
| Spc1 | CGTCGACGTTCGTGAATACATGT | *Sal*I | *spc^R^* |
| Spc2 | CCGGATCCGTTTTCTAAAATCTG | *Bam*HI | *spc^R^* |
| RA1 | GGATCCGCGCACAAGCAAGT | *Bam*H I | Downstream border of *htpsA* |
| RA2 | ACGGTACCTCAGGATGTTGCATGA | *Kpn* I | Downstream border of *htpsA* |
| Check1 | GGCTATGTGACATCACACGGTGA |  | Internal region of *divIVA* |
| Check2 | TCCCGTGTCTTCAATAACATCTGTC |  | Internal region of *divIVA* |
| Out1 | GAATGGGGAGAAATCAAACGAGTG |  | For combined PCR detection |
| Out2 | AACAAGATCCGTTGCTTCTCCATC |  | For combined PCR detection |
| **Real-time PCR** |  |  |  |
| 05SSU_0155-F | GTGACCAAATGGTTCTTGAC |  | *gapdh* |
| 05SSU_0155-R | ATTCAGTAGCAGCAGCTTTC |  | *gapdh* |
| 05SSU_0360-F | ACTGGACCAACTATGCCAAGGG |  | *galK* |
| 05SSU_0360-R | CCGTAAACAAAGACTTCCATCCCT |  | *galK* |
| 05SSU_0624-F | CGGAACCGTGAAACCTTGTATG |  | *arginine deiminase* |
| 05SSU_0624-R | AAAGAACCAATTCGTCACCACC |  | *arginine deiminase* |
| 05SSU_0626-F | ACCTTGGTGCCCATCCAGAATA |  | *arcB* |
| 05SSU_0626-R | AATACCGTCGAACATACGTCCC |  | *arcB* |
| 05SSU_0627-F | TCTGTTGTGACCCAGGTTATCG |  | *arcC* |
| 05SSU_0627-R | GCTTCTGCTTTCGCTTCTTCTT |  | *arcC* |
| 05SSU_0722-F | CTATGGTATCGGTGGCAAAGTC |  | *g*lpk |
| 05SSU_0722-R | GTTCCAATACGGAGCACCAAGT |  | *glpk* |
| 05SSU_0926-F | ATGTCTATCTGAAACCTGCTCTTGCC |  | *hypothetical protein* |
| 05SSU_0926-R | GATTTACCACCACCCAGCGAAC |  | *hypothetical protein* |
| 05SSU_0930-F | AAACGCCTTGACCTTGCCATTA |  | *transcriptional regulator* |
| 05SSU_0930-R | CTGCGGAAGACGGAGATACATT |  | *transcriptional regulator* |
| 05SSU_0931-F | GGGCTATGGCATGATGTTTGGC |  | *hypothetical protein* |
| 05SSU_0931-R | TGCGTGGACTGTCTGCTGTTGGAT |  | *hypothetical protein* |
| 05SSU_0962-F | GAACGGTTTGATTCGGATTGAG |  | *SNF2 family protein* |
| 05SSU_0962-R | TGCCTTTAGCTGATGAAGTGCC |  | *SNF2 family protein* |
| 05SSU_1013-F | TCTGTCATCCTACCTGGTGCTA |  | *glgC* |
| 05SSU_1013-R | ATATGGTCGATGTCTCCTGTGA |  | *glgC* |
| 05SSU_1153-F | GTCATAACAGATGCCAGCCACA |  | *β*-hexosamidase |
| 05SSU_1153-R | CGTTCGTCAGTAATCACTCCCT |  | *β*-hexosamidase |
| 05SSU_1159-F | ATGCGGGCTAATGGTATCTCAG |  | *uxaC* |
| 05SSU_1159-R | CTTCGGTCAACAATTCCTCAAC |  | *uxaC* |
| 05SSU_1219-F | TGGCTCGTATTGATGAGCGACTA |  | *PTS system* |
| 05SSU_1219-R | TGCCACGATGACAGTATTACAACCTA |  | *PTS system* |
| 05SSU_1403-F | CGAAATGGGATAACAGATGAGG |  | *s*ly |
| 05SSU_1403-R | TAAATGCGGCTTGAACTTGGGT |  | *s*ly |
| 05SSU_2123-F | TGGTCATCGGCTATGCTTTATC |  | *malM* |
| 05SSU_2123-R | CATCTTCCGTCACCTCATCCTC |  | *malM* |

^a^The underlined sequences are the restriction sites.
